# Label-Free 3D Visualization of Cellular and Tissue Structures in Intact Muscle with Second and Third Harmonic Generation Microscopy

**DOI:** 10.1371/journal.pone.0028237

**Published:** 2011-11-28

**Authors:** Markus Rehberg, Fritz Krombach, Ulrich Pohl, Steffen Dietzel

**Affiliations:** Walter-Brendel-Zentrum für Experimentelle Medizin, Ludwig-Maximilians-Universität München, München, Germany; Medical College of Georgia, United States of America

## Abstract

Second and Third Harmonic Generation (SHG and THG) microscopy is based on optical effects which are induced by specific inherent physical properties of a specimen. As a multi-photon laser scanning approach which is not based on fluorescence it combines the advantages of a label-free technique with restriction of signal generation to the focal plane, thus allowing high resolution 3D reconstruction of image volumes without out-of-focus background several hundred micrometers deep into the tissue. While in mammalian soft tissues SHG is mostly restricted to collagen fibers and striated muscle myosin, THG is induced at a large variety of structures, since it is generated at interfaces such as refraction index changes within the focal volume of the excitation laser. Besides, colorants such as hemoglobin can cause resonance enhancement, leading to intense THG signals. We applied SHG and THG microscopy to murine (*Mus musculus*) muscles, an established model system for physiological research, to investigate their potential for label-free tissue imaging. In addition to collagen fibers and muscle fiber substructure, THG allowed us to visualize blood vessel walls and erythrocytes as well as white blood cells adhering to vessel walls, residing in or moving through the extravascular tissue. Moreover peripheral nerve fibers could be clearly identified. Structure down to the nuclear chromatin distribution was visualized in 3D and with more detail than obtainable by bright field microscopy. To our knowledge, most of these objects have not been visualized previously by THG or any label-free 3D approach. THG allows label-free microscopy with inherent optical sectioning and therefore may offer similar improvements compared to bright field microscopy as does confocal laser scanning microscopy compared to conventional fluorescence microscopy.

## Introduction

Light microscopy is the method of choice for visualizing cells in their biological or physiological context. The classic light microscopy techniques such as Köhler illumination and phase contrast can be applied without prior labeling, allowing the observation of unperturbed cells or tissues. However, these techniques are not suitable for thick tissues and they do not allow high resolution three-dimensional reconstruction of image volumes, since the microscopic image also contains data from above and below the focal plane. This limitation can be circumvented by restricting image generation to photons originating from the focal plane as achieved by confocal laser scanning microscopy or by two-photon excited fluorescence microscopy. Both approaches require a fluorescent structure, and thus except for a few autofluorescent structures staining with structure specific fluorescent dyes such as DNA stains or fluorescent antibodies is necessary. Images from adjacent focal planes then can be combined to generate a three-dimensional image volume. For staining of living cells or tissues, however, it often remains unclear how far the staining perturbs physiologic functions of cells or tissues. For example, the expression of GFP or GFP fusion proteins was linked to induction of apoptosis [1], dilated cardiomyopathy in transgenic mice [2], impairment of actin-myosin interactions [3], [4], inhibition of polyubiquitination [5], and cytokine induction [6].

Second and Third Harmonic Generation (SHG and THG) microscopy [7], [8], [9] combine the advantages of optical sectioning and label-free visualization: Signal generation is restricted to the focal spot of the incoming laser while the need for potentially damaging fluorescent labeling is avoided. In the SHG process two simultaneously incoming photons are transformed into one emitted photon with exactly half the wavelength. SHG signals are generated at dense, non-centrosymmetric structures. In mammalian soft tissues, this equals mostly myosin in striated muscles and thick collagen type I, II and III fibers. In some special cases, dense microtubule bundles also can be visualized [10], [11], [12]. SHG microscopy is comparatively widespread, since detection is possible using standard two-photon microscopes with an excitation wavelength twice that of an active detection channel.

In THG, the energy of three simultaneously incoming photons is combined to emit one photon with one third the wavelength. THG is more complex to realize because detection of the signal in the visible range above 400 nm requires an excitation wavelength of above 1200 nm, a wavelength range not provided by standard two-photon microscopes. Detection in the visible range is not an absolute requirement dictated by physical theory, but rather by the optical elements of standard microscopes which have limited transmission for UV light. In addition, Rayleigh scattering with accompanying light loss in tissues increases with the 4^th^ power with shorter wavelengths [13]. THG microscopy is more versatile than SHG microscopy since THG is induced at interfaces such as refraction index changes within the focal volume of the excitation laser [8], [14]. Refraction indices change for example between cell nuclei and cytoplasm or cytoplasm and interstitial fluid. They are the basis for classical microscopy techniques such as phase contrast and DIC which are, however, less suitable for deep tissue 3D imaging since the contrast generating process is not limited to the focal plane. An additional source for THG which is of practical relevance in biological microscopy is resonance enhancement by light absorbing structures. For example hemoglobin induces a strong THG signal when illuminated with 1275 nm light, due to its absorption around 425 nm [14], [15], [16]. In summary, THG offers for unlabeled preparations what confocal and two-photon induced fluorescence did for fluorescent specimens, i.e. providing a technique with optical sectioning capability, with exclusion of out-of-focus signal and with 3D visualization.

While SHG and THG are well-established phenomena in the optical sciences, the biomedical community is less aware of their potential. To evaluate the capabilities of these label-free techniques, we applied THG and SHG microscopy to an established tissue model. The rodent cremaster muscle can be prepared from the scrotum as a thin, light permeable sheet and thus became widely-used for in vivo physiological research with conventional bright field microscopy (e.g. [17], [18], [19], [20]). In this well characterized tissue we investigated which structures were identified by SHG and THG and which level of detail could be achieved. The maximal achievable depth was determined in thigh muscles.

## Methods

### Specimen preparation

Tissue extraction was performed according to German legislation for the protection of animals. Male C57BL/6 mice at the age of 10 – 12 weeks were purchased from Charles River (Sulzfeld, Germany). Animals were housed under conventional conditions with free access to food and water. The Institutional Board of the Walter-Brendel-Zentrum has approved the tissue extraction after euthanization of the animals (approval ID K09), according to the local law "Bayerisches Tierschutzgesetz, Paragraph 6 Abs. 1 Satz 2 Nr. 4". The animals were euthanized by an intraperitoneal pentobarbital overdose (Narcoren, Merial, Germany). Subsequently, the right or left cremaster muscle was exposed through a ventral incision of the scrotum. The muscle was opened ventrally, and epididymis and testicle were detached, subsequently the cremaster was explanted. Throughout the procedure as well as after surgical preparation during microscopy, the muscle was immersed in buffered saline or 0.9% sodium chloride solution.

Structures and cells in isolated cremaster muscles were studied without fixation or other further treatment, except for those counterstained for DNA. For DRAQ5 staining, freshly excised, unfixed tissue was incubated for 15 – 60 min at room temperature in 0.9% NaCl with a 1∶1000 dilution of the 5 mM stock solution (Biostatus Ltd, Shepshed Leicestershire, UK). Microscopic observation was in 1∶10 diluted staining solution. For TO-PRO-3 (Invitrogen, Karlsruhe, Germany) staining, excised tissue was fixed for 10 min with buffered 4% paraformaldehyde (Microcos, Garching, Germany). Staining was for 1 hour in PBS with 2 µM TO-PRO-3 and 0.5% Triton X-100 (Sigma). TO-PRO-3-stained tissue was mounted in PermaFluor (Beckman Coulter, Fullerton, CA) on glass slides.

For imaging of unstained, unfixed cremaster preparations at room temperature, the muscle was placed on a standard microscopic glass slide or on a round cover slip and covered with sodium chloride solution (0.9%) solution. Round cover slips were mounted with vacuum grease (Baysilone-Paste mittel-viskös, GE Bayer Silicones GmbH & Co. KG, Leverkusen) in a plastic Petri dish with a hole in the bottom. On the sides, the edges of the muscle were weighed down to avoid movement of the tissue. Such cremaster muscle preparations have a thickness of 160 – 200 µm. For observation of leukocyte motility the tissue was placed in a custom-made heating device, submerged in Hank's Balanced Salt Solution and maintained at physiological temperature.

For the microscopy of thigh muscles, a longitudinal 10 mm incision through the skin of the right hind limb of a sacrificed mouse was made, beginning at the inguinal crease. The skin was removed to expose the musculus aductor magnus. The muscle was either directly immersed with saline, or excised and placed in a Petri dish filled with saline.

### Microscopy

Microscopic observations were performed using a commercially available system, a TriMScope (LaVision BioTec, Bielefeld, Germany) built around an Olympus BX 51 microscope (Olympus, Hamburg, Germany) and equipped with a tunable Ultra II Titanium:Sapphire Laser (Coherent, Dieburg, Germany) and an Optical Parametric Oscillator (Chameleon OPO; APE, Berlin, Germany; typical pulse width 200 fs, repetition rate 80 MHz) which is pumped by the Ti:Sa. The OPO generated 1270 nm or 1275 nm light with 640–700 mW output. The unattenuated intensity at the sample (i.e. after the objective) was ∼250 mW. Scanning cremaster tissue with full intensity and high resolution (400x400 µm with 1680×1680 pixels and 200 lines per second) did not produce any visible damage, except if the area of observation contained light absorbing dirt which would induce plasma formation. An Olympus XLUMPlanFl 20×/0.95W objective was used (working distance 2 mm). The following detection channels were used: backward (epi) detection blue (417 – 477 nm), red (604 – 644 nm) and far red (645 – 695 nm), forward detection blue and longpass 488 nm. 700 nm short pass filters blocked out excitation light. Light collection in forward direction was performed by an Olympus WI-UCD condenser, NA 0.8. Photomultiplier tubes (PMTs) were Hamamatsu H6780-01 for the blue channels and H6780-20 for the others. Where mentioned (see main text) more sensitive gallium arsenide phosphide detectors (Hamamatsu H7422-40) were used for backward detection.

To characterize the performance of the system we measured the full width half maximum (FWHM) of signals from subresolution particles embedded in 1% agarose with 1275 nm excitation. Two-photon excitation fluorescence of deep red beads (size 175 nm, exc 633, em. 660, Invitrogen P7220) provided an FWHM of 1.1 µm laterally and 6.2 µm axially. This compares to theoretical values of (0.51*λ) / ((√2)*NA)  = 484 nm laterally and (0.64* λ) / (n-√(n2-NA2))  = 1.7 µm axially [21], [22]. Latex beads did not generate a suitable THG signal. The THG-FWHM was measured with titanium dioxide nanoparticles (anatase configuration, <25 nm; Sigma-Aldrich 637254) which were dispersed with ultrasound in water as described [23]. For resulting particle aggregates an average size around 0.5 µm was described [23]. THG-FWHM was measured with 0.9 µm in xy and 4.5 µm in z. While the point-spread function (PSF) of two-photon excitation equals the squared illumination PSF [24], the three-photon excitation PSF equals the cubed illumination PSF and is therefore smaller. The smaller measured THG-FWHM is thus according to theoretical prediction. However, all values were far from diffraction limited. An optimization of the optics to 1275 nm should provide much narrower point spread functions and thus higher photon densities resulting in stronger harmonic signal generation.

### Image processing

Images were processed in ImageJ, Fiji and/or Imaris. ImageJ (W.S. Rasband, U. S. National Institutes of Health, Bethesda, Md, USA, http://imagej.nih.gov/ij/, 1997–2011) is a public domain 3D imaging package. Fiji (http://pacific.mpi-cbg.de/wiki/index.php/Fiji) is a distribution of ImageJ with a number of useful plugins already preinstalled. Imaris is a commercial visualization package from Bitplane, Zürich, Switzerland. Figures for publication were assembled in Adobe Photoshop. Images were adjusted for brightness but except for the obvious (cropping, scaling, etc.) not otherwise manipulated.

## Results and Discussion

### Collagen fibers and sarcomeres

**Figure 1:**
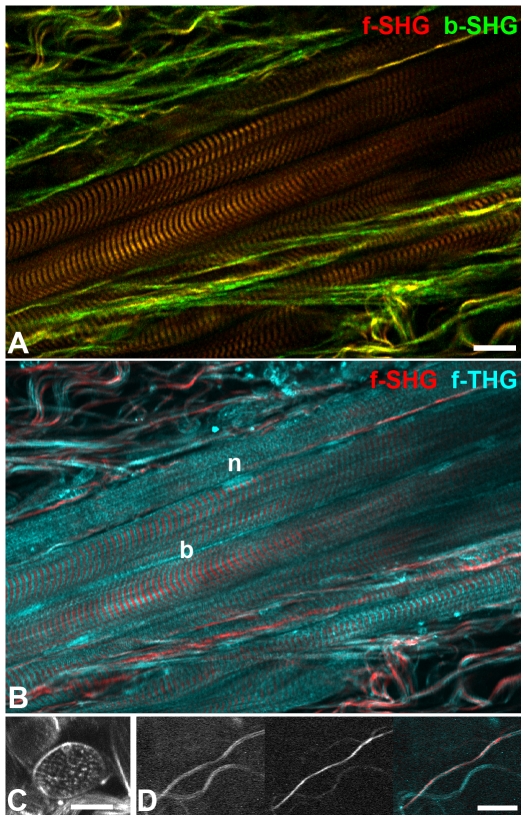
Collagen fibers and striated muscle. A) The relative SHG signal intensity ratio collagen/myosin is higher in backward SHG (green) than in forward SHG (red). B) While in striated muscle SHG visualizes myosin and thus A-bands of the sarcomeres, THG signals are from the interjacent I-bands as well as from the muscle fiber border (b) and muscle cell nuclei (n). C) Forward THG signal of a muscle fiber running parallel to the optical axis, single optical section. The fiber border is delineated by a surrounding THG signal. The small bubble at the upper fiber border may belong to a cell nucleus. No SHG signal was detected in this case. D) Thick collagen fiber producing a double signal in THG (left, cyan in overlay) which is filled by the SHG signal (center, red in overlay). Scale bars 20 µm.

Striated muscle and collagen fibers were inducers of SHG and THG (Figure 1a,b) and forward signals were generally stronger than backward signals, as described before [11], [25], [26], [27], [28]. As we found previously [28], in striated muscle THG and SHG signals are alternating (Figure 1b), arguing that THG inducing refraction index changes are largest in those parts of the sarcomeres that contain only actin. In addition, the outer membrane of muscle fibers induced THG, as did muscle fiber cell nuclei (Figure 1b, Movie S1). Muscle fibers running parallel to the optical axis produced dot-like THG signals in the interior, surrounded by a THG signal from the cell membrane (Figure 1c). Fibers at this orientation did not produce a detectable SHG signal, neither did muscle fibers inclined to the focal plane. Since higher harmonic generation depends on the relative spatial orientation of the incoming light waves to the specimen structure, we cannot deduce that the same structure is causing THG signals in horizontal and vertical muscle fibers.

For some collagen fibers, we observed a double signal with a hollow core of 1 – 1.5 µm (Figure 1d). This core was filled by the SHG signal, arguing that the fiber was wide enough to produce an independent THG signal (induced by refraction index mismatch) on each side. A similar phenomenon was observed by others in the rat aortic wall, where autofluorescence from elastic fibers was surrounded by parallel THG signals [29].

### Blood cells and vessels

**Figure 2:**
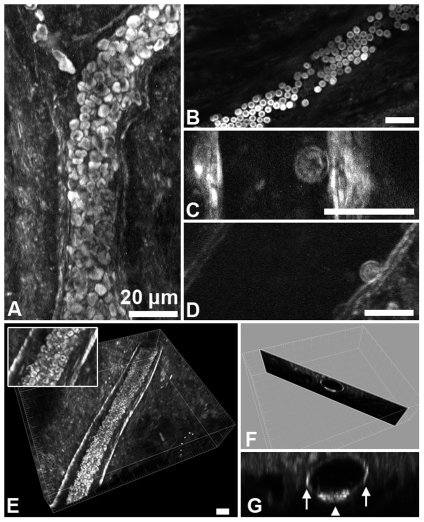
THG of blood vessels and blood cells in explanted, unfixed cremaster muscles recorded in forward direction. A) Projection of optical sections. Without flow, red blood cells (RBCs) sink to the bottom of the vessel, forming a tightly packed mass. B) Visualization of typical RBC shape. C,D) Leukocytes adhering to the inner vessel wall. E–G) Blood vessel with strong THG signal from the vessel wall. Vessel diameter is between 40 and 45 µm. E shows a 3D rendering with a 2x magnification in the inset. A section perpendicular to the vessel axis is shown in F and the same section is magnified in G. The arrows point to the prominent THG signal from wall sections parallel to the optical axis, the arrowhead points to the RBCs at the vessel bottom. All scale bars are 20 µm.

We visualized red blood cells (RBCs) in vessels of explanted, unfixed cremaster muscles, thus without flow, with forward detectors (Figure 2a). RBCs produced very strong signals and the spatial resolution was sufficient to reveal the biconcave shape typical for this cell type in the absence of external shearing forces under no flow conditions (Figure 2a,b,e). Individual RBCs [14], [16], [30] or blood [8], [31] were previously imaged with THG, but only very recently published images, recorded on a system similar to our own, provided sufficient optical resolution to suggest the typical biconcave shape [32]. In explanted tissue, RBCs sank to the bottom of the vessels and were densely packed in the lower vessel half (Figure 2g, arrowhead, Movie S1). Occasionally we observed leukocytes adhering to the inner vessel wall (Figure 2c,d).

Many blood vessels within the tissue were identifiable by THG not only by their RBC content but also by strong signals originating from the vessel walls (Figure 2e). Other blood vessels, however, did show only weak or no signal from the vessel walls (Movie S1). Whether these differences are due to local imaging conditions or whether they reflect differences between arterial and venous vessels will have to be investigated in future *in vivo* studies. If THG wall signals were present, they were emanating strongly from those wall segments running approximately in parallel to the optical axis (‘side walls’) while those segments parallel to the focal plane generated barely detectable or no signals (Figure 2e,g).

### Tissue-resident leukocytes

**Figure 3:**
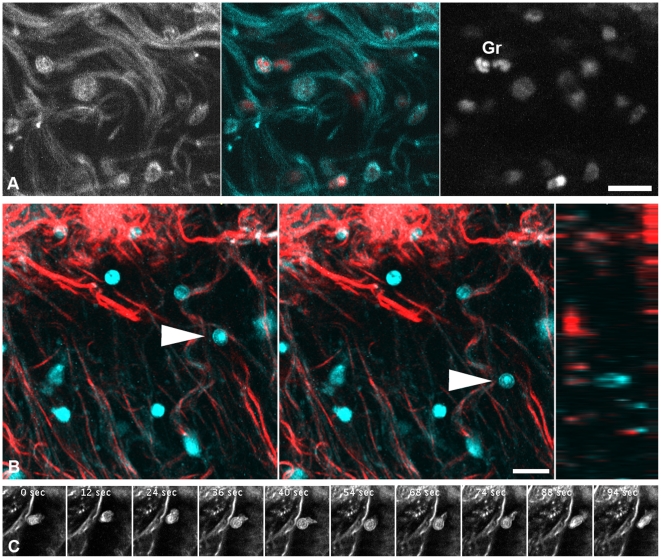
Migrating leukocytes. A) Fixed cremaster muscle, optical section about 50 µm from the surface. THG (left, cyan) revealed shapes reminiscent of migrating leukocytes deep in the tissue. DNA counterstaining (right, red) confirmed that these shapes are nucleated cells. In some cases, nuclei were lobed, a morphology typical for mature neutrophil granulocytes (Gr). Note that some cells identified by DNA stain are not visible in THG. B) Projections of THG (cyan) and SHG (red) in a time series of a freshly explanted cremaster, recorded in forward direction. While most leukocytes were stationary one moved ∼25 µm in 385 seconds (arrowheads). Image on the right shows a yz-section (view from the side, at end of the time series) illustrating that the moving cell is inside the tissue, not at the surface. The respective movie is shown in Movie S2. C) Migrating leukocyte in the freshly explanted cremaster forming and retracting pseudopodia. Single optical section. The respective movie is shown in Movie S3. Scale bars are 20 µm.

**Figure 4:**
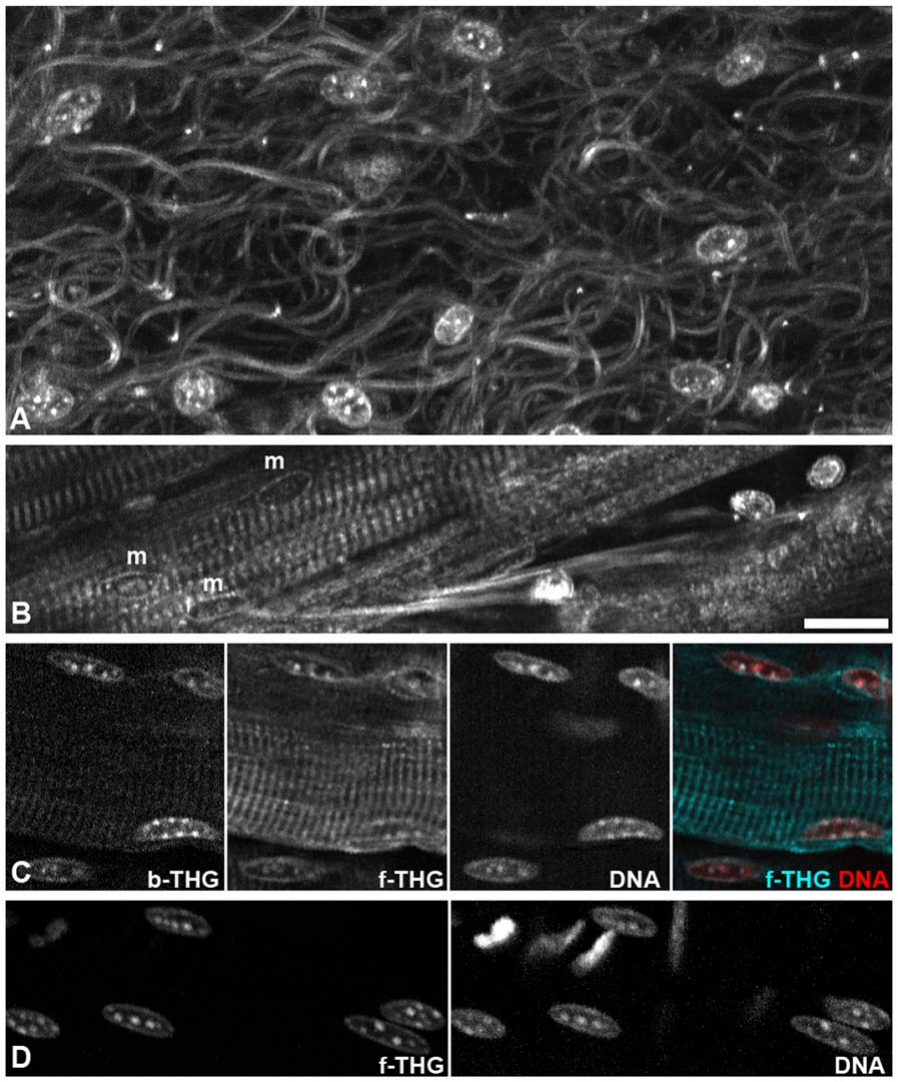
Nuclei and chromatin structure. A,B) Single optical forward THG sections from unstained, explanted cremaster. A, Section near the surface, nuclei most likely belong to macrophages. B, In deeper regions, nuclei of muscle fibers (m) can be observed, in addition to leukocytes. C,D) Nuclei in the cremaster after DNA-staining with Draq5. Chromocenters colocalize in THG channels and by DNA staining. C, Single optical section. D, Projections of five sections spaced 3 µm each. Not all DNA-stained-nuclei are visible in THG. Scale bar 20 µm for all images.

In the tissue, outside of blood vessels, we observed THG signals reminiscent of leukocytes. DNA staining after fixation confirmed that they were nucleated cells, with the shapes of some nuclei reminiscent of multi-lobed granulocyte nuclei (Figure 3a). THG imaging in unfixed tissue demonstrated movement over several dozens of micrometers and cellular dynamics of these cells with filopodia and lamellipodia extension and retraction events (Figure 3b,c; Movie S2, Movie S3). While movement of unlabeled leukocytes can also be observed by conventional transmission bright field microscopy or, with increased contrast, by reflected light oblique transillumination [33], to our knowledge, we here provide the first case of movement of live, unlabeled cells within tissues with three-dimensional resolution. Under good local imaging conditions, we found that even nuclear substructures can be recognizable not only in forward detected THG but also in backward detected THG (Figure 4c, see next paragraph). It thus should be possible to observe cellular migration also in solid organs, which can not be investigated by transmission microscopy.

### Nuclear organization

THG also visualized the nuclear chromatin structure in unfixed tissue (Figure 4a,b, Movie S1). The richness of detail depended on local imaging conditions and thus varied. Conditions with little scattering and absorption in the beam path were favorable, e.g. near the tissue surface but nuclei at some deeper sites also provided clear signals (Movie S1). As expected, THG signals recorded in forward direction generally provided better signal to noise ratios. However, for nuclei of muscle fibers running parallel to the focal plane, the chromatin structure was better discernable in backward recorded THG, since the strong forward THG signal from myosin obscured the details (Figure 4c). Apart from the THG signal generated at the nuclear border, we detected very bright internal structures. Some nuclei displayed up to eight such signals in one optical section, reminiscent in shape and number of chromocenters (Figure 4a,c,d, Movie S1). We therefore considered the possibility that chromocenters may generate THG signals. Chromocenters are large heterochromatic clusters, congregations of repetitive DNA, which are particularly prominent in mouse cell nuclei (see [34] and references therein). They were previously shown to have a higher refraction index than their nuclear environment [35]. Staining of unfixed cremaster tissue with the DNA dye Draq5 indeed revealed that the intranuclear THG signals colocalize with chromocenters (Figure 4c,d).

When excited with 266 nm, DNA shows autofluorescence with the maximum around 350 nm and extending up to around 425 nm [36], [37]. Our THG channels are equipped with detection filters transmitting light between 417 and 477 nm. To test for a contribution of multi-photon induced DNA autofluorescence to the observed chromatin signal, we removed the detection filter either in front of the forward detector or in front of the backward detector but not both. We then decreased the excitation wavelength from 1300 to 1200 nm in 10 nm steps while recording with both detectors. With detection filter the chromatin signal decreased dramatically at 1240 nm and was completely gone at 1230 nm and lower wavelengths while without filter the signal remained stable. This observation is in line with a THG signal at one third of the excitation wavelength. It is incompatible with a noteworthy contribution of fluorescence to the chromatin signal since the forward/backward intensity ratio of fluorescence should not change with the excitation wavelength.

To our knowledge, this is the first visualization of unlabeled chromocenters or any heterochromatin structure in living cells in 3D. The distribution of chromocenters changes during mouse cell differentiation to patterns characteristic for each cell type (see [34] and references therein) and relocation of centromeric heterochromatin was described during the cell cycle, e.g. in lymphocytes entering the cell cycle from a dormant stage (G_0_) upon activation [38], [39], [40]. Our finding that chromocenters induce THG opens the possibility to study the distribution of the these structures in living cells without the need for DNA stains which may interfere with nuclear function, a problem that surfaced with several respective dyes [41].

### Peripheral nerve fibers

**Figure 5:**
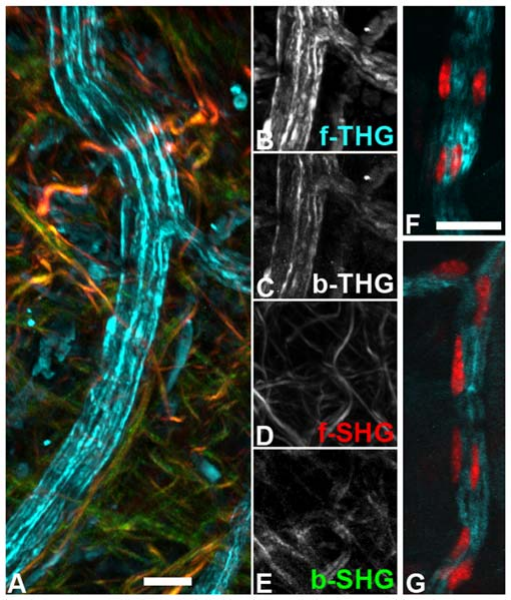
Nerve fibers in the cremaster. A) Unfixed, explanted muscle, projection of several optical sections covering 50 µm depth. Peripheral nerve (forward THG, cyan) surrounded by collagen fibers (forward SHG in red, backward SHG in green). Some blood vessels and small structures also produce THG signals. B–E) Single channel images of A near the nerve junction. Forward THG and SHG images have a much better signal to noise ratio than backward images. While forward and backward SHG signal differ substantially in some image details, backward THG (not included in A) does not contain any information in addition to the crisper forward THG image. F,G) Fixed cremaster with fluorescent DNA counterstain (red) and forward THG signal visualizing nerves. Several cell nuclei are associated with the nerve fibers. Scale bars are 20 µm, bar in F valid also for G.

The strongest THG signals we observed in the mouse cremaster muscle in forward as well as in backward direction were produced by peripheral nerve fibers (Figure 5). The strong backward-recorded signal opens the possibility to image peripheral nerve fibers also in tissues that do not allow collection of forward or reflected signals. We did not detect accompanying SHG signals as they were described for axonal microtubules in the rat central nervous system (CNS) [10], for nerve fibers in the zebrafish CNS [42] and in the mouse CNS [32].

As a potential source for the strong THG signal in nerve fibers, the packed membranes of the myelin sheath come to mind. Indeed, it was demonstrated earlier this year that nerve fibers in the mouse CNS also produce a strong THG signal and that this signal colocalizes with specific myelin staining [43]. In contrast to Farrar et al., however, we were not able to visualize the myelin sheath on top and at the bottom of the axons. Instead, as for blood vessels, we detected strong signals from the structures oriented in parallel to the optical axis. Given the large working distance of 2 mm of the objective used, THG provides the opportunity to visualize manipulators such as glass needles [32] simultaneously with huge or small nerves with high 3D resolution, providing direct optical control of the puncture site.

### Depth penetration

**Figure 6:**
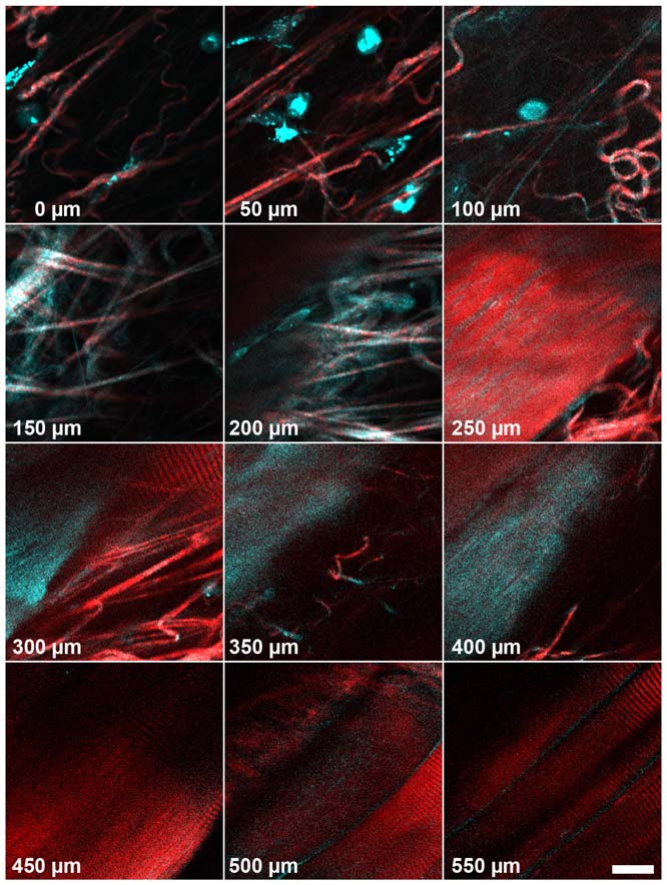
Deep tissue imaging in the mouse thigh muscle with backward GaAsP-detectors. The first 100 µm consist of connective tissue, followed by a dense meshwork of collagen fibers (150 and 200 µm) and finally striated muscle fibers with interspersed collagen. For this display, at each depth a different x,y position from the original 200×200 µm stack was selected. Each focal plane was adjusted for brightness individually to compensate for intensity differences. Scale bar is 20 µm.

For tissues substantially thicker than the mouse cremaster (150 – 200 µm), detection of transmitted forward generated SHG and THG signals is not an option. Instead, only backward detected signal can be obtained. While backward detection is hampered by a lower signal-to-noise ratio than forward detection, we did obtain backward detected SHG and THG signals through the whole depth of the cremaster. To estimate the depth until which backward signals can be recorded from thicker muscle tissue, we imaged mouse thigh muscles. A layer of connective tissue with a thickness of 100 – 250 µm was detected on top of striated muscle fibers (Figure 6). With our standard detectors, the maximum depth at which at least some structural information such as muscle striation or a collagen fiber was still recognizable varied with different regions of the tissue and was between 150 and 400 µm, with SHG on average 20 µm deeper than THG (compare Figure 6). We took advantage of an opportunity to test gallium arsenide phosphide (GaAsP) detectors instead of standard PMTs. Under identical excitation conditions, these more sensitive detectors allowed imaging depths up to 525 µm for THG and 550 for SHG (Figure 6).

The backward detected signals at a given distance from the surface may actually improve with increasing tissue thickness, since an increasing amount of the forward generated signal will be backscattered in deeper tissue layers. This assumption is supported by Légaré and colleagues showing that backscattered signal increased in intensity when a thin tissue section was put on top of a strongly scattering medium [44]. A recent report of backward THG imaging of the mouse brain up to 350 µm deep in the tissue also supports this view [32].

### Relation of forward and backward signals

As others [45], we found that forward to backward SHG intensity ratios varied for different structures. In our study in particular the forward/backward ratio was much higher for striated muscle fibers than for collagen fibers (Figure 1). This is in agreement with theoretical considerations [46] and earlier observations [12] that SHG inducing patterns, which are aligned to the incident light (along the optical axis) for a distance longer than the wavelength, create mostly forward SHG while shorter structures generate up to 50% backward SHG. Accordingly, the small subset of collagen fibers with stronger forward signal may be interpreted as having a better alignment with the polarization plane of the incident laser or a being thicker.

With THG, visualized structures did not usually show relative intensity differences for forward and backward detection. Backward THG generally appeared like a weak forward THG signal with decreased signal-to-noise ratio (Figure 5b,c and data not shown). Neither did a previous comparison of forward and backward THG describe structural differences between the images [47]. An exception to this rule was revealed by our comparison of forward/backward intensity ratios of chromocenters and muscle fiber striation. While images recorded in forward direction showed muscle striation and chromocenters in comparable intensity, backward images showed relatively intense chromocenters but weak striation (see above and Figure 4c). While backward THG signals were long thought to consist only of back scattered forward signal, a recent thorough theoretical and practical examination suggested that there is also a direct backward component. For structures thinner than 100 nm this backward component is relatively strong compared to the forward signal. The total amount of THG signal is, however, small for such a thin structure, since in the range of some hundreds of nanometers the THG signal decreases exponentially with decreasing thickness [48]. It is tempting to speculate that the relatively strong backward THG signal of chromocenters may be partly due to the high concentration of optically dense chromatin fibers immersed in less dense karyoplasm.

### THG as a tool in tissue imaging

A number of studies have established SHG as a tool to image collagen fibers and striated muscle myosin. More recently it becomes clear that THG may have an even broader potential for label-free three-dimensional microscopy of tissues. To our knowledge, this study is the first to demonstrate three-dimensional movement for unlabeled leukocytes within tissues (Figure 3b,c), optical sectioning of unlabeled chromatin structure (Figure 4) as well as the first visualization of unlabeled peripheral nerve fibers. Application of THG microscopy is thus the method of choice when staining of THG inducing structures is not possible or not desired, e.g. to avoid uncertainties about a non-physiological influence of dyes. In addition, since SHG and THG are coherent processes, they happen without energy deposition in the sample, in contrast to fluorescence excitation. Since SHG and THG are a direct consequence of the molecular composition of the sample, there is no bleaching of fluorochromes with the associated creation of free radicals and resulting phototoxicity, provided that simultaneous generation of autofluorescence is negligible [7], [8]. Instead, the high light intensities required for higher harmonic generation pose the risk of plasma formation, in particular at strongly absorbing structures such as melanin or other dark specks. Other important differences to fluorescence are the dependence of signal strength on the polarization direction of the exciting laser beam [27] and the uneven spatial distribution of the generated light, being generated mainly in forward direction [48], [49], [50].

While we have shown THG microscopy to provide resolution at a single cell level, it may be challenging to apply it to cells cultured directly on glass or plastic, since the medium/substrate interface generates a strong THG signal, raising the background for intracellular observations (data not shown).

Local imaging conditions are important also in other ways. The strength of SHG and THG signals depends heavily on the relative orientation of the potentially harmonophoric structure to the incoming polarized laser beam and on the optical conditions in the surrounding sample, such as absorption, scattering and internal refraction index mismatches in the beam path. For example, we observed shadowing by blood filled vessels on areas underneath. DNA staining revealed some cell nuclei that were not visualized by THG while others were visible (Figure 3a, Figure 4d). We obtained strong THG signals from those segments of blood vessel walls and myelin sheaths that are parallel to the optical axis, but not from those perpendicular although they are known to have the same molecular composition. Thus, the absence of a structure in a THG image does not necessarily equal the absence of the structure in the sample and respective interpretations should be made with caution. THG does, however, provide a wealth of three-dimensional information on unlabeled specimens not obtainable by other available techniques.

Two reasons argue for detection of THG signals in the visible range instead of generating UV-THG, e.g. with wavelengths available on Ti:Sa lasers (≤1060 nm): Transmission of the THG signal through typical glass optics is better in the visible range and the strong absorption and stronger scattering of UV light by biological tissues is avoided. Previous studies used a home-built Cr-Fosterite lasers emitting light at 1230 nm (e.g. [51], [52]) or other fixed wavelength lasers [14], [53] to achieve this goal. However, these light sources did not become widely used in biology labs. Thus, the possibility to perform THG microscopy was available only at very few locations. With the broader distribution of commercially available Optical Parametric Oscillators (OPOs) for multi-photon microscopy, this tunable light source is now also available for THG induction [48], [54]. These instruments, usually pumped by a Ti:Sa laser, are tunable and offer a pulsed, coherent beam with wavelengths from 1100 to 1300 nm and beyond. While most labs will acquire an OPO to allow two-photon fluorescence excitation of red fluorochromes with e.g. 1120 or 1180 nm, OPOs are also good light sources for THG imaging [55] as also shown in the current study. Given the advantages of label-free three-dimensional high resolution imaging, it is thus to be expected that THG microscopy will soon become a more common method for label-free 3D tissue imaging.

## Supporting Information

Movie S1 **Z-Sections through an explanted mouse cremaster muscle, forward THG signal.** Scale bar in lower left corner is 20 µm, the depth of each section is indicated in the lower right corner. The original 13 bit image (8192 gray values) was reduced to 8 bit (256 gray values) for movie generation. While visualizing less bright features, some of the brighter features became digitally overexposed during this process.
(AVI)

Movie S2 **Leukocyte moving in an explanted cremaster muscle.** While most leukocytes are stationary, one in the right half of the image is moving ∼25 µm in the 385 seconds of the observation period. Turning the image stack to the side demonstrates that the moving cell is indeed in the tissue, not on the surface. Forward THG signal in cyan, forward SHG signal in red. White scale bar is 20 µm.
(MP4)

Movie S3 **Leukocyte showing amoeboid movement.** Pseudopodia are extended and retracted during the process. The forward THG signal is shown.
(AVI)
